# Sequence-based prediction of SARS-CoV-2 vaccine targets using a mass spectrometry-based bioinformatics predictor identifies immunogenic T cell epitopes

**DOI:** 10.1186/s13073-020-00767-w

**Published:** 2020-08-13

**Authors:** Asaf Poran, Dewi Harjanto, Matthew Malloy, Christina M. Arieta, Daniel A. Rothenberg, Divya Lenkala, Marit M. van Buuren, Terri A. Addona, Michael S. Rooney, Lakshmi Srinivasan, Richard B. Gaynor

**Affiliations:** BioNTech US, Inc., 40 Erie Street, Suite 110, Cambridge, MA 02139 USA

**Keywords:** COVID-19, SARS-CoV-2 T cell epitopes, Computational biology, HLA-I binding prediction, HLA-II binding prediction, T cell assay, Vaccine design

## Abstract

**Background:**

The ongoing COVID-19 pandemic has created an urgency to identify novel vaccine targets for protective immunity against SARS-CoV-2. Early reports identify protective roles for both humoral and cell-mediated immunity for SARS-CoV-2.

**Methods:**

We leveraged our bioinformatics binding prediction tools for human leukocyte antigen (HLA)-I and HLA-II alleles that were developed using mass spectrometry-based profiling of individual HLA-I and HLA-II alleles to predict peptide binding to diverse allele sets. We applied these binding predictors to viral genomes from the *Coronaviridae* family and specifically focused on T cell epitopes from SARS-CoV-2 proteins. We assayed a subset of these epitopes in a T cell induction assay for their ability to elicit CD8^+^ T cell responses.

**Results:**

We first validated HLA-I and HLA-II predictions on *Coronaviridae* family epitopes deposited in the Virus Pathogen Database and Analysis Resource (ViPR) database. We then utilized our HLA-I and HLA-II predictors to identify 11,897 HLA-I and 8046 HLA-II candidate peptides which were highly ranked for binding across 13 open reading frames (ORFs) of SARS-CoV-2. These peptides are predicted to provide over 99% allele coverage for the US, European, and Asian populations. From our SARS-CoV-2-predicted peptide-HLA-I allele pairs, 374 pairs identically matched what was previously reported in the ViPR database, originating from other coronaviruses with identical sequences. Of these pairs, 333 (89%) had a positive HLA binding assay result, reinforcing the validity of our predictions. We then demonstrated that a subset of these highly predicted epitopes were immunogenic based on their recognition by specific CD8^+^ T cells in healthy human donor peripheral blood mononuclear cells (PBMCs). Finally, we characterized the expression of SARS-CoV-2 proteins in virally infected cells to prioritize those which could be potential targets for T cell immunity.

**Conclusions:**

Using our bioinformatics platform, we identify multiple putative epitopes that are potential targets for CD4^+^ and CD8^+^ T cells, whose HLA binding properties cover nearly the entire population. We also confirm that our binding predictors can predict epitopes eliciting CD8^+^ T cell responses from multiple SARS-CoV-2 proteins. Protein expression and population HLA allele coverage, combined with the ability to identify T cell epitopes, should be considered in SARS-CoV-2 vaccine design strategies and immune monitoring.

## Background

Coronaviruses are positive-sense single-stranded RNA viruses that have occasionally emerged from zoonotic sources to infect human populations [1]. Most coronavirus infections cause mild respiratory symptoms. However, some recent coronavirus infections have resulted in serious morbidity and mortality, including the severe acute respiratory syndrome coronavirus (SARS-CoV) [2–4], Middle East respiratory syndrome coronavirus (MERS-CoV) [5, 6], and SARS-CoV-2, which are responsible for the current worldwide pandemic, COVID-19. These three viruses belong to the genus *Betacoronaviridae* [1]. SARS-CoV was identified in South China in 2002, and its global spread led to 8096 cases and 774 deaths [7]. The first case of MERS-CoV emerged in 2012 in Saudi Arabia, and since then, a total of 2494 cases and 858 associated deaths have been reported [6]. In contrast to the more limited scope of these other coronavirus infections, SARS-CoV-2, which emerged in Wuhan, China, at the end of December 2019, has resulted in 9,129,146 cases, including 473,797 deaths globally as of June 24, 2020 [8]. The rapid spread of SARS-CoV-2 has resulted in the World Health Organization declaring a global pandemic. Thus, there is an urgent need for effective vaccines and antiviral treatments against SARS-CoV-2 to reduce the spread of this highly infectious agent.

The genome of SARS-CoV-2 spans 30 kb in length and encodes for 13 open reading frames (ORFs), including four structural proteins. These structural proteins are the spike protein (S), the membrane protein (M), the envelope protein (E), and the nucleocapsid protein (N). In addition, there are over 20 non-structural proteins that account for all the proteins involved in the transcription and replication of the virus [9]. All encoded proteins of the virus are potential candidates for developing vaccines to induce robust T cell immunity.

SARS-CoV and SARS-CoV-2 share 76% amino acid identity across the genome [10, 11]. This high degree of sequence similarity allows us to leverage the previous research on protective immune responses to SARS-CoV to aid in vaccine development for SARS-CoV-2 [12–15]. Both humoral and cellular immune responses have been shown to be important in host responses to SARS-CoV [16]. Antibody responses generated against the S and the N proteins have shown to protect from SARS-CoV infection in mice and have been detected in SARS-CoV and SARS-CoV-2-infected patients [17–20]. However, the antibody responses detected against the S protein were undetectable in patients 6 years post-recovery [21]. In addition, higher titers of antibodies have been found in more severe clinical cases of viral infection suggesting that a robust antibody response alone may be insufficient for controlling SARS-CoV [22] and SARS-CoV-2 [23–25] infection.

Together with B cell immunity, T cell responses seem important in the immune response’s control of SARS-CoV and are also likely important for the control of SARS-CoV-2. In mice, studies have shown that adoptive transfer of SARS-CoV-specific memory CD8^+^ T cells provided protection against a lethal SARS-CoV infection in aged mice and that adoptive transfer of effector CD4^+^ and CD8^+^ T cells to immunodeficient or young mice expedited virus clearance and improved survival [26]. Both CD4^+^ and CD8^+^ T cell responses have also been detected in SARS-CoV [16, 27] and SARS-CoV-2-infected patients [28–30]. Additionally, SARS-CoV specific memory CD8^+^ T cells have been found to persist for up to 11 years post-infection in patients who recovered from SARS [31]. These viral specific CD8^+^ T cells can be cytotoxic and can kill virally infected cells to reduce disease severity [16]. In addition to having effector functions, CD4^+^ T cells can promote the production of virus-specific antibodies by activating T-dependent B cells. Given the wealth of data from SARS-CoV, the homology between the SARS-CoV-2 and SARS-CoV, as well as emerging data from SARS-CoV-2 [28, 32], T cell immunity likely plays a critical role in providing protection against SARS-CoV-2.

Here, we utilized mass spectrometry (MS)-based HLA-I and HLA-II epitope binding prediction tools [33, 34] to identify SARS-CoV-2 epitopes recognized by CD4^+^ and CD8^+^ T cells. These binding predictors were trained on high-quality mono-allelic HLA immunopeptidome data generated via MS. The use of MS for the identification of MHC peptide ligandome yields an extensive and relatively unbiased population of naturally processed and presented MHC binding peptides in vivo. Unlike traditional binding assays which rely on chemical synthesis and a priori knowledge of peptides and ligands to be assayed, MS uses natural peptide-MHC complexes which are subject to the endogenous processing and presentation pathways within the cell. Additionally, the use of engineered mono-allelic cell lines avoids dependence on in silico deconvolution techniques and allows for allele coverage to be expanded in a targeted manner. In Abelin et al. [34], we demonstrated that improved HLA-II binding prediction led to improved immunogenicity prediction by validating this approach on a data set of immune responses to a diverse collection of pathogens and allergens [35, 36].

With this approach, we generated binding predictors for 74 HLA-I and 83 HLA-II alleles (Additional file 1: Table S1 and Additional file 2: Table S2). Alleles selected for data collection were prioritized to maximize population coverage. Here, we specifically validated the binding predictors utilizing *Coronaviridae* family peptides that had been assayed for T cell reactivity or MHC binding from the Virus Pathogen Resource (ViPR) database [34]. The ViPR database integrates viral pathogen data from internally curated data, researcher submissions, and data from various external sources. Specifically, experimentally determined epitopes were derived from the Immune Epitope Database (IEDB) [37]. Compared with a recent study with a similar aim [38], our approach provides epitope predictions to a wider set of alleles, which were characterized using high-quality mono-allelic MS data. These include high-frequency alleles from diverse populations as well as lower frequency alleles, leading to an expansive set of bioinformatically predicted SARS-CoV-2 epitopes.

We used our HLA-I and HLA-II binding predictors to predict the binding potential of peptide sequences from across the entire SARS-CoV-2 genome for a broad set of HLA-I and HLA-II alleles, covering the vast majority of USA, European, and Asian populations (Additional file 3: Table S3). We additionally confirm that a subset of these epitopes can raise specific CD8^+^ T cell responses in T cell induction assays using donor PBMCs. Furthermore, we interrogate publicly available proteomic data and demonstrate that the relative expression of SARS-CoV-2 proteins in virally infected cells vary significantly, pointing to another parameter that should be considered in vaccine design to induce cellular immunity. Epitopes predicted to have a high likelihood of binding to multiple HLA-I and HLA-II alleles and exhibit high expression in infected human cells are promising vaccine candidates to elicit T cell responses against SARS-CoV-2.

## Methods

### Analysis of *Coronaviridae* family T cell epitopes from ViPR

Experimentally determined epitopes for the *Coronaviridae* family for human hosts were retrieved from the ViPR database (https://www.viprbrc.org/; accessed March 5, 2020) [39]. To build a validation dataset, both positives and negatives for T cell assays and MHC binding assays were obtained. Only assays associated with alleles identified with at least four-digit resolution and supported by our predictors (Additional file 1: Table S1) were included in this analysis. Positive calls were prioritized: peptide-allele pairs were classified as positive if a given peptide-allele pair was assayed multiple times by a specific assay type and was determined to be positive in any single one of the assays. Specifically, the priority was given by the following order: Positive-High > Positive-Intermediate > Positive-Low > Positive > Negative (e.g., a peptide allele pairing that was assayed three times with the results Positive-High, Positive, and Negative were assigned a Positive-High result). Of note, alternative approaches such as prioritizing negative assay results, or random choice in cases of multiple results, yielded very similar results.

### Binding prediction for ViPR *Coronaviridae* family T cell epitopes

Peptide-HLA-I allele pairs in the ViPR validation dataset were scored using our HLA-I binding predictor, a neural network trained on mono-allelic MS data [33]. Similarly, peptide-HLA-II allele pairs in the ViPR validation dataset were scored using our HLA-II binding predictor, a recently published convolutional neural network-based model also trained on mono-allelic MS data [34]. We scored all 12–20mers contained within a given assay peptide with the HLA-II binding predictor and took the maximum score as the representative binding score for the assay peptide. In vitro MHC binding assays, which represent the vast majority of the ViPR dataset, do not require endogenous processing and presentation for a positive binding result. Since our binding predictor, which is trained on naturally processed and presented ligands observed via MS, is also implicitly learning these endogenous processing rules, we score all potential ligands within an assayed peptide (rather than just the full-length assay peptide itself) to account for this distinction.

### Retrieval of SARS-CoV-2 sequence

The GenBank reference sequence for SARS-CoV-2 (accession: NC_045512.2, https://www.ncbi.nlm.nih.gov/nuccore/NC_045512) was used for this study. All twelve annotated open reading frames (ORF1a, ORF1b, S, ORF3a, E, M, ORF6, ORF7a, ORF7b, ORF8, N, and ORF10) were considered as sources of potential epitopes. In addition, due to its high expression level in recently published proteomic datasets [40–42], ORF9b, as annotated by UniProt (P0DTD2, https://www.uniprot.org/proteomes/UP000464024), was also used for epitope predictions.

### Identification of HLA-I epitopes and prioritization by population coverage

To identify candidate HLA-I epitopes, we exhaustively scored all possible 8–12mer peptide sequences from SARS-CoV-2 with our HLA-I binding predictor [33] for 74 alleles, including 21 HLA-A alleles, 35 HLA-B alleles, and 18 HLA-C alleles. Peptide-allele pairs were assigned a percent rank by comparing their binding scores to those of 1,000,000 reference peptides (selected from a partition of the human proteome that had not been used for model training) for the same respective allele. Peptide-allele pairs that scored in the top 1% of the scores of these reference peptides were considered strong potential binders.

Since a vaccine should ideally benefit a large fraction of the population, these top-ranking peptides were then prioritized based on expected population coverage (allele frequencies obtained from [43]), given all the alleles each peptide was expected to bind to (i.e., all the alleles for which the peptide scored in the top 1%). The estimate of population coverage for each peptide was calculated as:

$$ \mathrm{Coverage}=1-{\Pi}_{\mathrm{loci}}\ {\left(1-{\Sigma}_{\mathrm{locus}\ \mathrm{alleles}}\ {f}_{\mathrm{allele},\mathrm{avg}}\right)}^2 $$

where *f*_allele,avg_ is the (unweighted) average allele frequency across the US, European, and Asian-Pacific Islander (API) populations (which is intended to represent an approximation of a global population average, focusing on the populations most affected by the pandemic, and the cumulative product is taken across the three HLA-I loci: HLA-A, HLA-B, and HLA-C). The cumulative product itself represents the chance that an individual in the population does not express any one of the contained alleles; hence, the complement describes the probability that at least one is present.

The USA population allele frequency is calculated as the following weighted average of a few subpopulations: 0.623*EUR + 0.133*AFA + 0.068*API + 0.176*HIS, where EUR = European, AFA = African American, API = Asian-Pacific Islander, and HIS = Hispanic populations. These subpopulation frequencies are based on data from the US Census Bureau [44], accommodating for slight variations in different tables and information of mixed races. For alleles where AFA, HIS, or API population frequencies were not available, the US population allele frequency values were set to match EUR. Missing API allele frequency values were conservatively imputed with 0 for our analyses.

We then constructed two types of ranked lists of HLA-I epitopes by coverage. The first ranks all SARS-CoV-2 epitopes by their absolute coverage, such that peptides predicted to bind similar collections of alleles would be ranked similarly (Additional file 4: Table S4). This approach provides the full list of predicted class I epitopes sorted by the expected coverage for each peptide, with the generous assumption that every binding prediction is correct.

The second type of list, referred to as a “disjoint” list, is constructed in an iterative fashion where the peptide with the greatest coverage is selected first, and then, the coverage for the remaining epitopes is updated to nullify contributions from any alleles that have already been selected (Additional file 5: Table S5). Disjoint lists were generated for M, N, and S proteins (the most highly expressed structural proteins) individually, instead of across the entire SARS-CoV-2 genome, to provide protein-level prioritizations. This approach produces a parsimonious list of peptides that is designed to maximize cumulative population coverage with the fewest number of selections.

### Identification of HLA-II epitopes and prioritization by population coverage

To identify HLA-II epitopes, we used our HLA-II binding predictor [34] to score all 12–20mer sequences in the SARS-CoV-2 proteome to predict both binding potential and the likely binding core within each 12–20mer. Scoring was performed across all supported HLA-II alleles, consisting of 46 HLA-DR alleles, 17 HLA-DP alleles, and 20 HLA-DQ alleles (Additional file 2: Table S2).

Peptide-allele pairs were assigned a percent rank by comparing their binding scores to those of 100,000 reference peptides (as before, sampled from a partition of the human proteome that was held out from training). Pairs scoring in the top 1% were deemed likely to bind. Additionally, we define the “epitope” of 12–20mers to be the predicted binding core within the sequence. As such, overlapping 12–20mers with the same predicted binding core for a given allele would constitute a single epitope.

**Table 1:** Summary of the HLA-I and HLA-II epitopes predicted across the 13 SARS-CoV-2 ORFs and their validation

ORFs	Length (AA)	Peptide HLA-I pair count	Reported in ViPR	Assay: Negative	Assay: Positive	Percent—positive	Binding-core and HLA-II pair count
Envelope protein (E)	75	556	34	3	31	91.2	29
Membrane glycoprotein (M)	222	1236	41	0	41	100.0	68
Nucleocapsid phosphoprotein (N)	419	1054	40	9	31	77.5	107
ORF1a polyprotein*	4405	14*	0	0	0	NA	0*
ORF1ab polyprotein	7096	28,965	0	0	0	NA	2516
ORF3a protein	275	1408	127	11	116	91.3	94
ORF6 protein	61	322	0	0	0	NA	23
ORF7a protein	121	642	3	0	3	100.0	28
ORF7b	43	327	8	1	7	87.5	2
ORF8 protein	121	449	20	2	18	90.0	27
ORF9b protein**	97	453	6	1	5	83.3	37
ORF10 protein	38	258	0	0	0	NA	4
Spike protein (S)	1273	4686	95	14	81	85.3	437

*Peptides unique to ORF1a (not found in ORF1ab)

**Annotated in UniProt

Additionally, we prioritized predicted HLA-II binding 25mers in SARS-CoV-2 by population coverage, given the desire to design vaccines that are effective broadly across the global population. To do this, we associated each 25mer with all subsequences that were likely binders and calculated the population coverage of the corresponding HLA-II alleles. Given a collection of alleles, we calculated the coverage as described in the previous section, the only difference being the cumulative product is taken across the following four HLA-II loci: HLA-DRB1, HLA-DRB3/4/5, HLA-DP, and HLA-DQ. HLA-II allele frequencies were obtained from [43] and Allele Frequency Net Database [45].

As with HLA-I, two types of sorted lists of predicted binding sequences were generated. The first type ranks every predicted SARS-CoV-2 25mers by absolute coverage provided by the HLA-II alleles to which a constituent subsequence is expected to bind (Additional file 6: Table S6). The second type of ranking was again performed for predicted binders in M, N, and S proteins individually, using disjoint coverage, to maximize cumulative population coverage with a parsimonious list of peptides (Additional file 7: Table S7).

### Comparison of predicted epitopes to the human proteome

Eight to 12mer sequences (corresponding to predicted HLA-I epitopes), 9mer sequences (corresponding to predicted HLA-II binding cores), and 25mer sequences (corresponding to predicted HLA-II sequences that bound multiple alleles) from SARS-CoV-2 were compared against all subsequences of the same length from the human proteome, using UCSC Genome Browser genes with hg19 annotation of the human genome and its protein coding transcripts (63,691 entries) [46]. Exact matches were identified, flagged in Additional file 4: Table S4, and omitted from the disjoint coverage ranking analysis to avoid prioritizing peptides that may inadvertently induce an autoimmune response. No exact matches were found for the predicted HLA-II binding cores or 25mer sequences.

### T cell inductions and assessment of peptide-MHC-positive T cell responses

Human PBMCs from HLA-A02:01-positive human donors were isolated using Ficoll separation from apheresis material (AllCells, USA). Twenty-three SARS-CoV-2 peptides predicted to be strong binders to HLA-A02:01 were pooled by similar binding potential, with up to 6 peptides per pool. The selected peptides represent high ranking peptides predicted to bind HLA-A02:01 from across the S, N, M, E, and ORF1ab proteins, avoiding sequences also prioritized by Grifoni et al. [38]. Specifically, three of the 23 peptide sequences were chosen from ViPR and also scored highly in our predictions; the remaining 20 were the top-ranking peptides by our prediction for the abovementioned proteins. When comparing our 20 SARS-CoV-2-predicted epitopes with the ViPR dataset, 8 epitopes were previously assayed and confirmed as HLA-A02:01 binders. Of these 8, two were reported positive in a T cell assay in ViPR and two were negative. PBMCs were incubated with peptide pools, matured, and cultured in the presence of IL-7 and IL-15 (CellGenix GmbH, Germany) to promote T cell growth. Cells were then harvested, and the frequency of CD8^+^ T cells specific to peptide-MHC (pMHC) was assayed using combinatorial coding of pMHC multimers [43].

pMHC multimers were prepared as described previously [47, 48]. Briefly, biotinylated HLA-A02:01 monomers loaded with UV cleavable peptides were exchanged under UV light with SARS-CoV-2-predicted peptides. The streptavidin-labeled fluorophores PE, APC, BV421 (Biolegend, Inc., USA), BV650, and BUV395 (BD Biosciences, USA) were added to UV-exchanged monomers to create fluorescently labeled multimer reagents.

Harvested cells were then stained with LIVE/DEAD Fixable Near-IR Dead Cell Stain Kit for 633 or 635 nm excitation (Life Technologies Corporation, USA); anti-CD4 FITC, anti-CD14 FITC, anti-CD16 FITC, and anti-CD19 FITC (BD Biosciences, USA); and anti-CD8 AF700 (Biolegend Inc., USA). Only live CD8^+^ T cells staining for both fluorochromes of the relevant pMHC multimers were considered positive [47]. Samples were analyzed on FACS LSR Fortessa X20 cytometers (BD Biosciences), and data was analyzed using FlowJo (TreeStar).

### Analysis of publicly available SARS-CoV-2 proteomic datasets

SARS-CoV-2 proteomic datasets were downloaded from the PRIDE repository (Bojkova et al. [40]: PXD017710, https://www.ebi.ac.uk/pride/archive/projects/PXD017710; Bezstarosti et al. [41]: PXD018760, https://www.ebi.ac.uk/pride/archive/projects/PXD018760; Davidson et al. [42]: PXD018241, https://www.ebi.ac.uk/pride/archive/projects/PXD018241). In these studies, either Caco-2 human colorectal adenocarcinoma cells [40] or Vero E6 African green monkey kidney epithelial cells [41, 42] were subject to infection with SARS-CoV-2. Tandem mass spectra (MS/MS) acquired with data-dependent acquisition (DDA) were interpreted using Spectrum Mill MS Proteomics software package v7.0 pre-release (Agilent Technologies). Cysteine carbamidomethylation was selected as a fixed modification. Methionine oxidation, asparagine deamidation, protein N-termini acetylation, peptide N-terminal glutamine to pyroglutamic acid, and peptide N-terminal cysteine pyro-carbamidomethylation were selected as variable modifications. For the dataset from Bojkova et al. [40] which employed isobaric mass tags, TMT11 was added as a fixed modification to peptide N-termini and lysines, and ^13^C_6_-^15^N_2_-TMT11-lysine and ^13^C_6_-^15^N_4_-arginine were added as variable modifications. All datasets were searched against the SARS-CoV-2 proteome (UniProtKB, 28 April 2020, 14 entries) concatenated to databases containing either the *Homo sapiens* proteome ([40], UCSC Genome Browser hg19 annotation, 63,691 entries) or the *Chlorocebus sabaeus* proteome ([41, 42] UniProtKB, 9229 entries). Precursor and fragment mass tolerances were set as described in each manuscript, or as 20 ppm when not specified. Database search results were exported as a list of peptide-spectrum matches (PSMs) with a target-decoy-based false discovery rate (FDR) estimation of 1%. Individual fractions from each study were combined into a single list. To perform spectral counting, PSMs assigned to a single SARS-CoV-2 protein were counted, with ORF1a and ORF1ab treated as a single protein group. Peptides matched to both a host and SARS-CoV-2 protein were discarded. Spectral counts were normalized to the length of each protein, and the maximum value within each dataset was set to 100%.

## Results

### Bioinformatics predictor validation for viral epitopes using ViPR

We first sought to validate the ability of our predictors to identify epitopes from genomes of the *Coronaviridae* family. Since SARS-CoV-2 only emerged recently, specific data on SARS-CoV-2 peptide MHC binding and immunogenic epitopes are currently limited. However, other viruses from the *Coronaviridae* family have been studied thoroughly, specifically MERS-CoV and SARS-CoV. The latter has significant sequence homology to SARS-CoV-2 [12]. We therefore sought to leverage previously tested epitopes from across the *Coronaviridae* family to validate our predictions of viral peptides, with special interest in peptide sequences that exactly matched protein sequences of the novel SARS-CoV-2 virus. To that end, we used the ViPR database [39], which lists the results of T cell immunogenicity and MHC-peptide binding assays for both HLA-I and HLA-II alleles for viral pathogen epitopes. We used all assays of *Coronaviridae* family viruses with human hosts from ViPR as our validation dataset. Assays that did not have an associated four-digit HLA allele or were associated with an allele our models did not support were omitted (see Additional file 1: Table S1 and Additional file 2: Table S2 for a list of supported alleles).

For HLA-I, within the validation dataset, there were a total of 4445 unique peptide-HLA allele pairs that were assayed for MHC binding, using variations of (1) cellular MHC or purified MHC, (2) a direct or competitive assay, and (3) measurement by fluorescence or radioactivity. Two additional peptide-MHC allele pairs were confirmed via X-ray crystallography. Depending on the study from which the data was collected, peptide-MHC allele pairs were either defined in ViPR simply as “Negative” and “Positive” for binding, or with a more granular scale of positivity: low, intermediate, and high. We assigned peptide-MHC allele pairs with multiple measurements with the highest MHC binding detected across the replicates (see the “Methods” section).

**Fig. 1:**
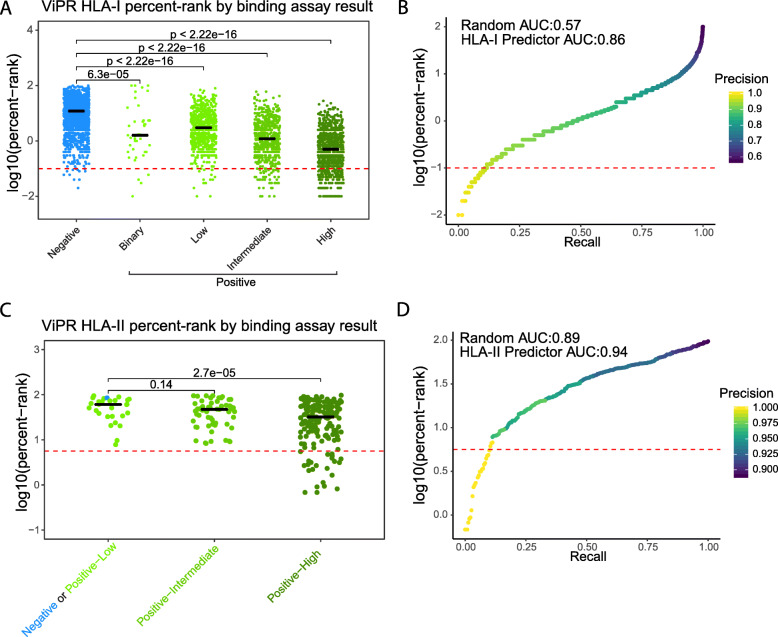
Binding predictions for both peptide-HLA-I and HLA-II pairs from ViPR correlate with their reported assay results. **a** The log10(percent rank) of scored peptide-HLA-I allele pairs, versus their ViPR reported MHC binding assay result (either binary Negative/Positive or the scaled Negative/Positive-Low/Positive-Intermediate/Positive-High, based on the reported value). In total, there were 4445 peptide-HLA-I allele pairs in the ViPR dataset we obtained (see the “Methods” section). Black lines indicate median values. **b** A modified precision-recall analysis for the HLA-I binding prediction of ViPR data, in which we demonstrate the fraction of the true positives out of all called positives (precision, indicated by color) and the fraction of detected true positives out of all true positives (recall, indicated *x*-axis value) as a fraction of the log10(percent-rank) threshold (*y*-axis value). Red dashed line indicates an example of log10(percent-rank) threshold of − 1, corresponding to the line in **a**. Area under the precision-recall curve is indicated. **c** The log10(percent rank) of scored peptide-HLA-II allele pairs, versus their ViPR reported MHC binding assay result (Negative+Positive-Low/Positive-Intermediate/Positive-High, based on the reported value). In total, there were 259 peptide-HLA-II allele pairs. Black lines indicate median values. **d** A modified precision-recall analysis for the HLA-II binding prediction of ViPR data, in which we demonstrate the fraction of the true positives out of all called positives (precision, indicated by color) and the fraction of detected true positives out of all true positives (recall, indicated *x*-axis value) as a fraction of the log10(percent-rank) threshold (*y*-axis value). Red dashed line indicates an example of log10(percent-rank) threshold of − 1, corresponding to the line in **c**. Area under the precision-recall curve is indicated

We then applied our HLA-I binding predictor to the peptide-MHC allele pairs in the validation dataset and compared the computed HLA-I percent ranks of these pairs with the reported MHC binding assay results (Additional file 8: Table S8). A low percent rank value corresponds to high likelihood of binding (e.g., a peptide with a percent rank of 1% scores among the top 1% in a reference set of random peptides). The percent ranks of peptide-MHC allele pairs that had a binary “Positive” result in the MHC binding assay were significantly lower than pairs with a “Negative” result. Further, in the more granular positive results, stronger assay results (low < intermediate < high) were associated with increasingly lower percent ranks (Fig. 1a). In addition, the two peptide-MHC alleles that were confirmed by X-ray crystallography were predicted as very likely binders, with low percent rank scores of 0.07% and 0.30%. Although our HLA-I binding predictor was initially built with the purpose of supporting neoantigen prediction in cancer, this analysis shows that it can be successfully applied to coronavirus proteomes. We evaluated our predictor by performing a Precision-Recall analysis, demonstrating the tradeoff between accurate calling of positive binders and the fraction of true binders that are detected (Fig. 1b).

Assays of T cell reactivity (e.g., interferon-gamma ELISpots, tetramers), which are stricter measures for T cell immunogenicity to epitopes, were performed in significantly lower numbers compared with MHC binding assays. For HLA-I, the overlap between peptide-MHC allele pairs for which we had a prediction (supported alleles) and pairs with a reported T cell assay consisted of only 32 pairs, of which 23 had a positive result. We did not detect differences in the percent ranks across the positive and negative groups; however, sample sizes are extremely small. In addition, for HLA-I epitopes, the validation dataset only contained T cell assay results for peptide-MHC allele pairs that had a positive result in a binding assay, suggesting a highly biased pool of epitopes selected for testing, as also reflected in the high rate of positive T cell assay results. Indeed, the high rate of positive MHC binding assays compared to what would be expected for completely randomly selected peptides also implies that peptides expected to bind based on prediction or prior data were prioritized for testing (or negative results were under-reported). This underlying bias in peptides assayed is important to keep in mind in evaluating the binding predictor performance on this validation dataset. An even more dramatic difference in scores for positives versus negatives could be expected had random peptides been selected for testing.

In addition to the identification of targets for CD8^+^ T cells, we have recently demonstrated the ability to predict HLA-II binders [34], allowing us to target CD4^+^ T cell responses which could be harnessed for SARS-CoV-2 vaccines. These CD4^+^ responses can potentially bolster both T cell immunity and enhance humoral immunity [49].

In a similar fashion to the HLA-I analysis, we scored all *Coronaviridae* family peptide-MHC allele pairs with supported HLA-II alleles in ViPR using our HLA-II binding predictor [34] (Additional file 9: Table S9). There were 259 unique peptide-MHC allele pairs assayed by MHC binding assays in the ViPR validation dataset for HLA-II. As before, we compared their percent rank with their reported “best” (in the case of multiple measurements) MHC binding assay result. This comparison could not be performed with the “Negative” pairs as an independent group since there was only one negative result in the validation dataset for HLA-II. The low negative counts may be due to under-reporting of negative assay results or biased selection of the peptides to be assayed. Therefore, we merged the “Negative” and “Positive-Low” groups into one group and compared their percent ranks with either the “Positive-Intermediate” or the “Positive-High” groups (Fig. 1c). This analysis revealed a trend similar to that observed with HLA-I predictions, indicating that stronger MHC binding assay results are associated with a lower predicted percent rank for HLA-II binders, as we expect for a robust predictor. We also evaluated our HLA-II binding predictor by performing a precision-recall analysis (Fig. 1d). The area under the precision-recall curve (AUC) indicated only a small advantage to our predictor over a random guess, which is explained by the heavy bias towards peptides with positive HLA-II binding assay results. Similar to the HLA-I T cell assays, there were too few recorded HLA-II T cell assays in our validation dataset to determine percent rank differences between peptide-HLA II allele pairs testing positive and negative. Together, these findings further corroborate the validity of our epitope predictors, as peptide-MHC allele pairs with positive results in binding assays consistently have lower percent ranks (better scores) by both our HLA-I and HLA-II MHC binding predictors.

### Epitope prediction for SARS-CoV-2

**Fig. 2:**
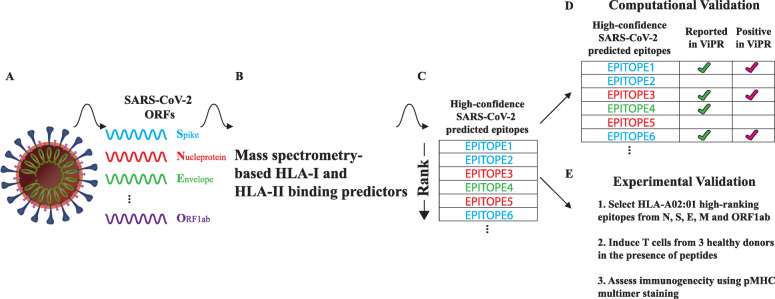
A schematic demonstrating our approach to identify SARS-CoV-2 T cell epitopes and their validation. (**a**) A diagram of the SARS-CoV-2 virus, listing example proteins. (**b**) Applying our HLA-I and HLA-II binding predictors to the 13 annotated ORFs of SARS-CoV-2 (including ORF9b). (**c**) Both HLA-I and HLA-II epitopes are ranked by their likelihood to bind a particular HLA allele. (**d**) Epitopes shared between SARS-CoV-2 and other coronaviruses which were previously assayed are used for validation. (**e**) A description of a T cell induction assay to assess immunogenicity of select epitopes

We harnessed our HLA binding prediction ability to identify the peptides most relevant to the generation of SARS-CoV-2 T cell responses. We first performed the analysis for HLA-I peptide binding and computed the likelihood of each peptide of lengths 8–12 amino acids from the 13 SARS-CoV-2 ORFs to bind to any HLA-I allele in our database. We then calculated the percent rank of each peptide-MHC allele pair by comparing their binding scores to those of a set of reference peptides; putative binders were identified as sequences predicted to bind to a given allele with a percent rank of 1% or lower (Fig. 2 a–c).

By this metric, we detected a total of 11,897 unique SARS-CoV-2 peptides that were predicted to bind at least one HLA-I allele (Additional file 4: Table S4). Sixteen of these peptides overlapped with a subsequence of the human proteome and were marked for considerations of potential autoimmunity (see the “Methods” section, Additional file 4: Table S4).

Unlike HLA-I, which has a closed binding groove that constrains bound peptide lengths to approximately 8 to 12 amino acids, peptides binding HLA-II have a wider length distribution (up to 30 amino acids or even longer) since the HLA-II binding groove is open at both ends. Peptides bind with a 9-amino acid subsequence (termed the binding core) occupying the HLA-II binding groove, with any flanking sequence overhanging the edges of the molecule. We consider a group of peptides that differ in the flanking regions but share a common binding core as a single epitope. Using the HLA-II predictor, we identified 3372 unique binding cores that are predicted to bind at least one HLA-II allele with a percent rank score of 1% or lower (Table 1). The majority of predicted peptide-MHC allele pairs are from ORF1a and ORF1ab, primarily driven by the length of these ORFs. In addition, ORF1a and ORF1ab have very similar sequences, with over 18,000 identical binding peptide-HLA-I allele pairs predicted for both ORFs. We therefore opted to exclude redundant predictions and only reported unique pairs (see * in Table 1). Similarly, all HLA-II predicted epitopes from ORF1a were covered by those reported for ORF1ab.

To test the validity of the SARS-CoV-2-predicted peptide-HLA pairs, we looked for peptide sequences in the *Coronaviridae* portion of the ViPR database which exactly matched SARS-CoV-2 peptide sequences (Fig. 2d). A total of 374 HLA-I peptide-MHC allele pairs from SARS-CoV-2 had both a percent rank lower than 1% by our predictor and were found in the HLA-I MHC binding validation dataset. Strikingly, of these HLA-I peptide-MHC allele pairs, 333 (89%) had a positive assay result. As a comparison, we also tested for overlap between epitopes predicted to have low likelihood of MHC binding (percent rank 50% or higher) and the validation dataset. Thirty-seven peptide-MHC allele pairs overlapped between these sets, of which 36 (97.2%) had a negative assay result, as predicted. Further, we sought to determine whether our highly predicted SARS-CoV-2 peptide-HLA-I allele pairs (percent rank lower than 1%) would be validated by reported T cell assay results. Despite the significantly smaller number of peptide-MHC allele pairs that were tested for T cell reactivity in the validation dataset, 10 assayed pairs were also highly predicted by our HLA-I binding predictor. Nine out of these 10 (90%) predicted pairs had a positive result to the T cell assay. No low-scoring pairs (percent rank of 50% or above) were reported in the validation dataset. These findings demonstrate the validity of our prediction for peptide-HLA-I allele pairs for SARS-CoV-2 epitopes. Notably, while our algorithms are not trained on T cell reactivity data and are aimed at peptide-MHC binding, for the few examples in ViPR for which T cell reactivity assay results were reported, we were able to show our highly scoring peptide-MHC allele pairs are indeed immunogenic in the vast majority of cases.

For HLA-II peptide-MHC allele pairs, only a single HLA-II peptide-MHC allele pair had both a percent rank lower than 1% and was reported in the validation dataset; this single pair (from the envelope protein) had a “Positive-High” assay result.

### Immunogenicity of HLA-A02:01-predicted SARS-CoV-2 epitopes

**Fig. 3:**
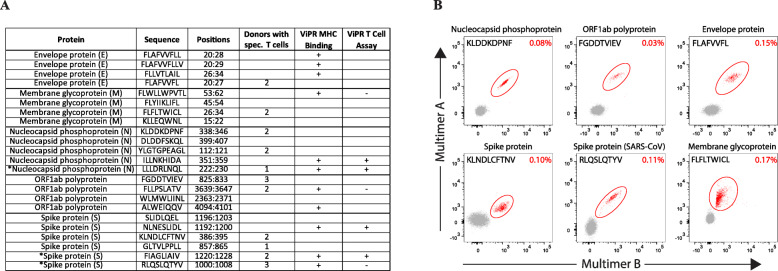
Experimental Validation of HLA-A02:01 predicted epitopes from SARS-CoV-2 in human T cell induction assays. **a** 23 peptides that were predicted to be high binders to HLA-A02:01 were synthesized and assayed in T cell inductions using PBMCs from three human donors. Three epitopes marked with asterisk were chosen based on ViPR, while the remaining 20 were chosen solely based on the predictor score. For our assay, epitopes were considered to be immunogenic if at least one donor raised a T cell response to the peptide as determined by pMHC multimer technology. ViPR confirmation refers to identical sequences from SARS-CoV confirmed via either MHC binding or T cell assays. **b** Flow cytometry plots of pMHC multimer staining from representative immunogenic SARS-CoV-2 epitopes. Multimer positive populations are circled in red, with the frequency of multimer positive CD8^+^ T cells shown in the upper right-hand corner of each plot

Our binding prediction algorithms predict the likelihood of an epitope to be presented by a specific HLA allele, but do not directly predict the ability of a T cell receptor to recognize the epitope presented by the MHC molecule. Due to the process of central tolerance, which deletes T cells that could cross-react with peptides from self-antigens, not every epitope that is a strong MHC binder will elicit a T cell response [50]. Therefore, there is a need to further validate high affinity MHC binding peptides in T cell assays as previously described (Fig. 2e) [51–54]. To address the immunogenicity of a subset of highly predicted MHC binding peptides, we synthesized 23 highly predicted HLA-A02:01 binding epitopes from each of the following SARS-CoV-2 proteins: S, M, N, E, and ORF1ab (Fig. 3a). Of these, 20 were selected solely due to being highly predicted SARS-CoV-2 epitopes while the additional three are also highly predicted but were chosen from ViPR. Pools of these peptides were cultured with PBMCs from three human donors, and the predicted epitopes were considered immunogenic if they elicited a T cell response as detected by binding to pMHC multimers for HLA-A02:01 in at least one of three donors.

Overall, we detected CD8^+^ T cell responses in at least one donor for 11 of the 23 highly predicted epitopes in our assay (Fig. 3a, b). Fifteen of the 20 epitopes (75%) selected solely based on the prediction score were either reported positive in ViPR for MHC binding (*n* = 8) or had T cell reactivity in our assay (*n* = 8) in association with HLA-A02:01. Importantly, 10 of these 20 predicted epitopes (50%) tested positive for T cell reactivity, either in our assay (*n* = 8) or in previous reports of SARS-CoV (*n* = 2), confirming that our binding predictor can identify epitopes that are immunogenic. We were thus able to identify eight novel epitopes not previously reported in ViPR that were recognized by specific CD8^+^ T cells in donor PBMCs. The responses were generally robust, with nine of the 11 epitopes positive in our assay being recognized by specific CD8^+^ T cell responses in at least two donors (Fig. 3a), and encouragingly, every ORF from SARS-CoV-2 that was assayed had at least one peptide that led to a T cell response (Fig. 3a). Taken together, these data show that many novel SARS-CoV-2 epitopes that were predicted to be strong binders from our HLA-I binding predictor were found to be immunogenic.

### Population coverage of peptides predicted to bind multiple HLA-I and HLA-II alleles

**Fig. 4:**
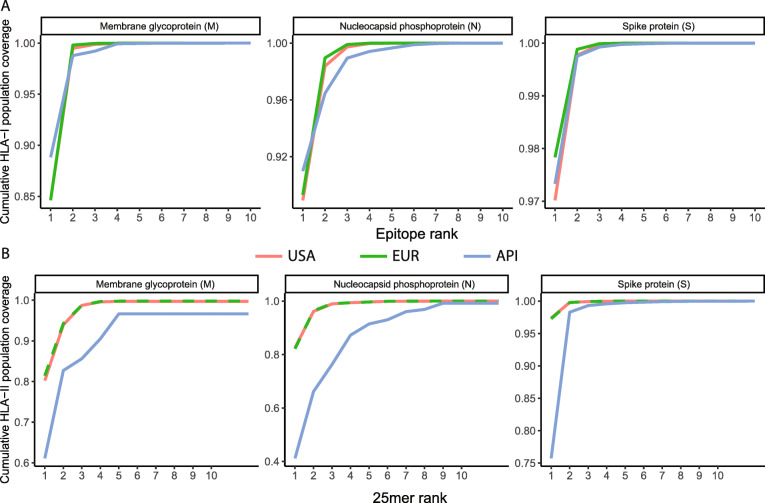
Few predicted multi-allele binding epitopes from individual SARS-CoV-2 proteins can achieve broad population coverage. **a** Cumulative HLA-I coverage for USA, EUR, and API populations versus the number of included prioritized HLA-I epitopes for M, N, and S proteins, respectively. See Additional file 5: Table S5 for the peptide sequences corresponding to each panel. **b** Cumulative HLA-II coverage for each population versus the number of included prioritized HLA-II 25mers for M, N, and S proteins, respectively. See Additional file 7: Table S7 for the peptide sequences corresponding to each panel

We sought to prioritize peptides from the M, N, and S proteins that are predicted to provide broad coverage for the US, European, and Asian-Pacific Islander populations based on the prevalence of MHC alleles in these populations [43]. We found that a subset of the peptides was predicted to bind a broad set of either HLA-I or HLA-II alleles. For each protein, we determined that a small number of peptide sequences provide saturating coverage for the US, European, and Asian-Pacific Islander populations, with > 99% population coverage achieved with selected 8–12mer epitopes for HLA-I, and > 95% population coverage achieved with selected 25mer sequences for HLA-II, respectively (Fig. 4a, b). Even if the generous assumption that all peptide-MHC allele pairs for which a given peptide scores in the top 1% are indeed immunogenic is not fully upheld, this finding could facilitate the design of a parsimonious, broadly effective vaccine to induce broad T cell immunity.

### Leveraging proteomic data to infer relative viral protein abundance

**Fig. 5:**
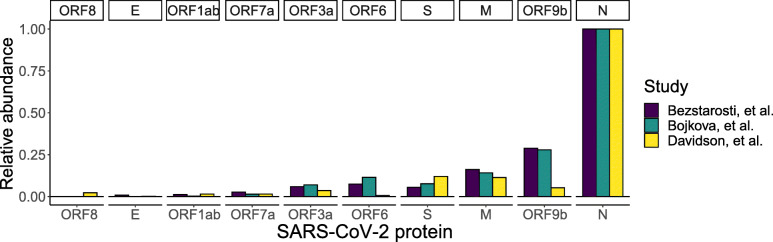
Analysis of publicly available proteomic datasets demonstrates relative SARS-CoV-2 protein expression levels. Three datasets examining the proteomic response to SARS-CoV-2 infection were re-analyzed, and protein abundance was estimated by spectral counts normalized to protein length. Any annotated ORF not shown in the figure was not detected in these proteomic studies. Across all three studies, the nucleocapsid protein (N) is the most abundant SARS-CoV-2 protein in infected cells

In addition to peptide-MHC binding, another important consideration in the design of a potential SARS-CoV-2 vaccine is the degree of viral protein expression in infected host cells. In order to determine the relative abundance of SARS-CoV-2 proteins, we analyzed three publicly available proteomic datasets that acquired unbiased LC-MS/MS on tryptic digestions of SARS-CoV-2-infected host cells [40–42]. Relative abundance of the viral proteins was estimated by spectral counting, a semi-quantitative approach whereby peptide-spectrum matches are counted, and totals are compared across proteins (Additional file 10: Table S10) [55, 56]. This analysis demonstrated the significantly wide range of expression levels of the SARS-CoV-2 proteins. Specifically, it confirmed that the N protein is the most abundant viral protein across all three datasets following SARS-CoV-2 infection (Fig. 5). This finding is corroborated by reports of N-derived peptides being detected in gargle solution samples from COVID-19 patients [57]. Furthermore, the N protein has been used as a biomarker for diagnosing patients infected with the SARS-CoV virus [58]. On the other hand, based solely on genomic information, ORF10 might be considered a potential target for vaccine development. However, there is very little proteomic and transcriptomic evidence that ORF10 is actually expressed in SARS-CoV-2-infected cells [42, 59]. These findings emphasize the value of considering SARS-CoV-2 protein expression levels in addition to HLA binding predictions and the immunogenicity of these epitopes in vaccine design strategies.

## Discussion

In this work, we demonstrated the utility and validity of our HLA-I and HLA-II binding prediction algorithms to the *Coronaviridae* virus family, and specifically to SARS-CoV-2. We use our validated predictors trained on mono-allelic MS data for both HLA-I and HLA-II binders, which potentially could be leveraged to induce both long-term CD4^+^ and CD8^+^ T cell immunity against the virus. Specifically, our HLA-II predictor, which has been trained on a large set of mono-allelic MS data and has been shown to identify immunogenic epitopes, is used here to identify high-quality SARS-CoV-2 CD4^+^ epitopes that may contribute to both cellular and humoral immunity [34] (Additional file 6: Table S6). Our database of supported HLA-I and HLA-II alleles provides us with the ability to not only identify many peptide-MHC allele pairs, but to generate a narrow list of peptides with many potential HLA pairings that could be presented by the entire US, European, and Asian-Pacific Islander populations. By applying these algorithms to previously assayed peptide-MHC allele pairs in ViPR, we were able to demonstrate excellent concordance between our binding predictions and the results of the binding assays for both HLA-I and HLA-II epitopes. We leveraged the homology within the *Coronaviridae* family to demonstrate that an exceedingly high portion (~ 90%) of our high-ranking SARS-CoV-2 peptide-MHC allele pairs for which validation was available was indeed confirmed to bind the predicted MHC allele.

We also experimentally confirmed that our binding predictors can identify epitopes that are immunogenic and can lead to CD8^+^ T cell responses to multiple SARS-CoV-2 proteins in donor PBMCs. It is plausible that the significant fraction of epitopes we experimentally confirmed (50% of highly predicted, tested epitopes) is only an underestimate for overall immunogenicity, since PBMCs from only three donors were used in this initial experiment. While an immunogenicity rate of 50% for epitopes predicted based on HLA binding is encouraging, it is challenging to compare with previous studies due to differences in T cell induction protocols, prediction algorithms and their prediction thresholds, and epitope etiology [53, 60, 61]. Pre-prints released during the revision of this manuscript [60, 61] studied CD8^+^ T cell responses to a small set of epitopes from the S protein in COVID-19 patient or healthy donor PBMCs. The study by Shomuradova et al. [60] showed that in their cohort, following stimulation, two HLA-A02:01 epitopes (YLQPRTFLL and RLQSLQTYV) distinguished CD8^+^ T cell responses in COVID-19 patients from healthy donor samples collected before or during the pandemic. Their data also show that 10 additional peptides sporadically induce responses in 2–3 individuals from either the healthy donor or the COVID-19 patient cohorts (total of 31 individuals). We found that 11 of the 12 peptides are highly ranked by our predictor (percent rank < 1%), with the two peptides specifically immunogenic in patients scoring at 0.1 and 0.17% rank, respectively. Interestingly, in our T cell assay, the HLA-A02:01 epitope RLQSLQTYV elicited a CD8^+^ T cell response in all three healthy donors.

While focusing on a small set of two COVID-19 patients and one healthy donor, Chour et al. [61] demonstrate that five HLA-A02:01-restricted epitopes from the S protein elicited a CD8^+^ T cell response in all three individuals. Although these epitopes were not tested in our immunogenicity assay, all five (100%) were predicted highly by our HLA-I binding predictor (percent rank < 1%). These studies provide independent confirmation to our algorithm’s ability to not only predict HLA-I binding, but also identify immunogenic peptides.

Though we did not perform T cell assays to evaluate the immunogenicity of the HLA-II predicted epitopes, such analysis would be valuable, especially given the importance of CD4^+^ T cells in both the cellular and humoral anti-viral response. We thus propose that a combination of B and T cell epitopes could provide long-lasting immunity from SARS-CoV-2 or mitigate the severity of disease when protection is partial.

We therefore concluded that using our HLA binding predictors to predict T cell epitopes from the ORFs of SARS-CoV-2 provides a novel and large set of high-quality T cell vaccine targets for the virus. In comparison to the recent publication by Grifoni et al. [38], we provide a large number of predicted epitopes, which we attribute to two differences in our approaches: (a) we opted to use a less stringent prediction cutoff based on the concordance of our 1% rank cutoff with the previously reported epitopes in ViPR and (b) we provide predictions for alleles from a wider range of population frequencies. This approach better covers non-white populations and provides predictions for rare alleles based on models trained on mono-allelic MS data as opposed to the extrapolation required by pan-allele predictors for alleles lacking sufficient data. This approach allows us to better prioritize vaccine candidates, as well as provide researchers with predictions to investigate materials from individuals with less frequent alleles. The differences in training data lead to algorithms that prioritize substantially different epitope sets, even for well-studied alleles. For instance, of the 91 HLA-A02:01 epitopes nominated by Grifoni et al., only 48 overlap with the top 91 from our predictor. In addition, we provide not only bioinformatics validation based on previously reported T cell epitopes and MHC binding peptides from other viruses from the *Coronaviridae* family in ViPR, but also experimentally validated, novel SARS-CoV-2 T cell epitopes.

The selection of target sequences can be further guided by levels of protein expression, predicted population coverage, and degree of sequence conservation. First, designing therapeutics against predicted epitopes is only effective if the proteins containing those epitopes are expressed at high enough levels for efficient antigen processing and presentation to take place. Therefore, it is crucial that protein expression be considered when selecting therapeutic targets. Second, prioritization of epitopes that are predicted to bind multiple alleles could provide coverage to significant fractions of the population, while including few epitopes in the vaccine. Lastly, during the viral spread and expansion through the population, genomic modifications are acquired, generating sequence diversity among the SARS-CoV-2 variants. This diversity may allow evasion of immune pressure, and therefore, it is important to prioritize epitopes that are conserved across the SARS-CoV-2 variants [62]. Novel tools which enable restricting peptides to conserved regions have recently become available: https://covidep.ust.hk [63] and http://cov-glue.cvr.gla.ac.uk/#/home [64].

Limiting epitope selection to highly expressed proteins, epitopes predicted to bind multiple high-frequency HLA alleles, and conserved viral sequences restricts the number of potential epitopes. However, the breadth of the list we provide increases the likelihood of identifying many high-quality, highly expressed epitopes. The epitopes characterized here, combined with insights on SARS-CoV-2 protein expression along with further efforts to confirm immunogenicity, can provide pre-clinical validation of epitopes that may be vaccine candidates to induce strong cellular immunity.

## Conclusions

In summary, our work provides the most extensive set of both CD4^+^ and CD8^+^ T cell epitopes that are spanning the entire SARS-CoV-2 genome and binding a wide set of HLA-I and HLA-II alleles. Combining this epitope list with consideration of protein expression levels, population coverage and viral sequence conservation will lead to generation of a short list of vaccine epitope candidates that are likely immunogenic in the majority of the population. Our predicted list of CD4^+^ and CD8^+^ T cell epitopes will complement B cell epitopes and serve as a resource for the scientific community to generate potent SARS-CoV-2 vaccine epitopes and generate long-lasting T cell immunity.

## Supplementary information

**Additional file 1: Table S1.** HLA-I Alleles Covered by Binding Predictors. List of the 74 alleles covered by the HLA-I binding predictor. (CSV 896 bytes)

**Additional file 2: Table S2.** HLA-II Alleles Covered by Binding Predictor. List of the 83 alleles covered by the HLA-II binding predictor. (CSV 1 kb)

**Additional file 3: Table S3.** HLA-I and HLA-II Allele Population Frequencies. HLA-I and HLA-II allele frequencies for USA, European (EUR), Asian Pacific Islander (API), African American (AFA), and Hispanic (HIS) populations.

**Additional file 4: Table S4.** Predicted HLA-I Binders Ranked by Population Coverage. Table of all predicted HLA-I binders and their associated allele coverage, including additional indicators for overlap with the human proteome or overlap with the ViPR dataset used. (CSV 1448 kb)

**Additional file 5: Table S5.** Broadly Binding HLA-I Peptides. The top HLA-I predicted binders from each of the three SARS-CoV-2 proteins: spike, nucleocapsid and membrane with the broadest cumulative allele coverage. (CSV 19 kb)

**Additional file 6: Table S6.** SARS-CoV-2 25mers Ranked by HLA-II Population Coverage. Table of all SARS-CoV-2-derived 25mers containing at least 3 predicted HLA-II binders as subsequences. (CSV 1268 kb)

**Additional file 7: Table S7.** Broadly Binding HLA-II 25mers. The top HLA-II predicted binders from each of the three SARS-CoV-2 proteins: spike, nucleocapsid. (CSV 16 kb)

**Additional file 8: Table S8.** binding prediction of ViPR HLA-I epitopes. The peptide-HLA alleles pairs from the ViPR database which belong to the *Coronaviridae* family and have a human host that had been scored using our HLA-I binding predictor. (CSV 522 kb)

**Additional file 9: Table S9.** binding prediction of ViPR HLA-II epitopes. The peptide-HLA alleles pairs from the ViPR database which belong to the *Coronaviridae* family and have a human host that had been scored using our HLA-II binding predictor. (CSV 39 kb)

**Additional file 10: Table S10.** spectral counts from published SARS-CoV-2 proteomic datasets. MS/MS spectra assigned to peptides from SARS-CoV-2 proteins were tallied across datasets, divided by protein length, and normalized within each dataset to generate Fig. 5.

**Additional file 11.** Custom Python script for HLA-I. This Python scripts can be used to generate HLA-I supplementary tables. (PY 5 kb)

**Additional file 12.** Custom Python script for HLA-II. This Python scripts can be used to generate HLA-II related supplementary tables. (PY 8 kb)

**Additional file 13.** Custom R script for figure plotting. This R script can be used to plot the precision-recall analyses from Fig. 1, reproduce Fig. 5, and to produce Table 1. (R 9 kb)

## Data Availability

All data generated or analyzed during this study are included in this published article, its supplementary information files, or the following external sources: SARS-CoV-2 reference sequences used in this study were obtained from GenBank (accession: NC_045512.2, https://www.ncbi.nlm.nih.gov/nuccore/NC_045512) and ORF9b, as annotated by UniProt (P0DTD2, https://www.uniprot.org/proteomes/UP000464024). The method has been described in depth in Abelin et al., Immunity 2017 [33] and Abelin et al., Immunity 2019 [34]. Analogous online tools to the ones deployed here are available at (a) http://hlathena.tools/ for HLA I epitopes which is maintained by the Broad institute, and described in Sarkizova et al., Nature Biotechnology 2019 alongside with the associated data [65], and (b) https://neonmhc2.org/ for HLA II epitopes [34]. Mono-allelic MS data used for the training of our HLA-II binding predictor is also available as part of Abelin et al., Immunity 2019 [34]. SARS-CoV-2 proteomic datasets were downloaded from the PRIDE repository (Bojkova et al. [40]: PXD017710, https://www.ebi.ac.uk/pride/archive/projects/PXD017710; Bezstarosti et al. [41]: PXD018760, https://www.ebi.ac.uk/pride/archive/projects/PXD018760; Davidson et al. [42]: PXD018241, https://www.ebi.ac.uk/pride/archive/projects/PXD018241). Custom R and Python scripts used in generation of supplementary tables and figures are included (Additional files 11, 12 and 13).

## Abbreviations

HLA: Human leukocyte antigen
MERS-CoV: Middle East respiratory syndrome coronavirus
MHC: Major histocompatibility complex
MS: Mass spectrometry
PBMC: Peripheral blood mononuclear cells
PSM: Peptide-spectrum matches
SARS-CoV: Severe acute respiratory syndrome coronavirus
SARS-CoV-2: Severe acute respiratory syndrome coronavirus 2
USA: United States of America
ViPR: Virus Pathogen Resource
WHO: World Health Organization
